# Alternating current electroluminescent fibers for textile displays

**DOI:** 10.1093/nsr/nwac113

**Published:** 2022-06-14

**Authors:** Zhen Wang, Xiang Shi, Huisheng Peng

**Affiliations:** State Key Laboratory of Molecular Engineering of Polymers, Department of Macromolecular Science, and Laboratory of Advanced Materials, Fudan University, China; State Key Laboratory of Molecular Engineering of Polymers, Department of Macromolecular Science, and Laboratory of Advanced Materials, Fudan University, China; State Key Laboratory of Molecular Engineering of Polymers, Department of Macromolecular Science, and Laboratory of Advanced Materials, Fudan University, China

## Abstract

This perspective summarizes the research status of textile displays based on fiber-shaped and interwoven light-emitting devices with remaining challenges and future directions.

With the emerging 5G and Internet of Things, wearables are developing vigorously [1]. Wearables are expected to be flexible, lightweight, breathable and comfortable to satisfy daily applications [2]. Integrating electronic devices into textiles to form an electronic textile (e-textile) will simultaneously meet the above requirements and the need for daily clothes. E-textiles with functions of communicating, sensing and supplying power have been widely explored. As the main output terminal of electronic devices, display can provide a revolutionary human–computer interaction experience when integrating with e-textile. Flexible thin-film displays are traditionally fixed onto the surfaces of textiles, in order to integrate displays with textiles (Fig. 1a). However, the structure and mechanical mismatch between thin-film display and textile lead to not only the loss of breathability and softness of textile, but also unstable light emission when the device is severely deformed [3]. As the basic unit of textile, fibers are more suitable and flexible to be integrated into textiles compared with their traditional planar counterparts (Fig. 1b).

**Figure 1.**
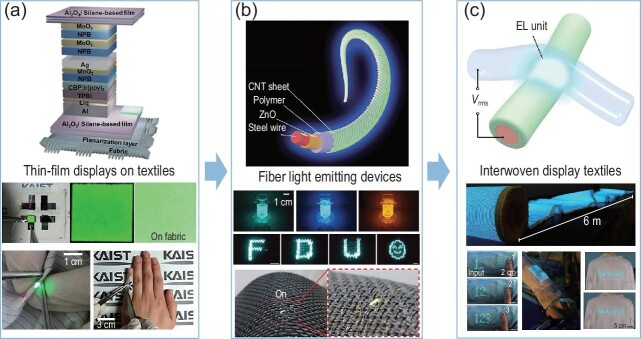
The evolution of displays on textiles and their representative configurations. (a) Thin-film displays on textiles [3]. (b) Fiber light-emitting devices [4,5,7]. Copyright (2018) by the American Chemical Society. (c) Interwoven textile displays [9].

Active luminescent materials are the core of fiber light-emitting devices that mainly determine the design and performance of these devices. According to the classification of luminescent materials, they can be categorized as organic light-emitting diode (OLED), polymer light-emitting diode (PLED), light-emitting electrochemical cell (LEC) and alternating current electroluminescent (ACEL) fibers (Table 1).

**Table 1.** Technical comparisons among OLED, LEC and ACEL fibers.

Light-emitting fibers	OLED fibers [4,10,11]	LEC fibers [5,6]	ACEL fibers [7–9]
Mechanism	Carrier injection	Carrier injection	Electric field activation
Material	Conjugated small molecules or polymers	Light-emitting polymerelectrolyte, ionic transition metal complexes	Sulfide materials such as ZnS, SrS, CaS
Fabrication	Vacuum deposition, dip-coating	Dip-coating, wrapping	Dip-coating and extrusion
Brightness	High (∼10 000 cd/m^2^)	High (∼1000 cd/m^2^)	Low (100–300 cd/m^2^)
Length	Centimeters	Centimeters	Meters
Interface requirement	High	Medium	Low (physical contact)
Working mode	Pattern display and pixel display	Pattern display	Pattern display and pixel display

OLED fibers share a typical coaxial architecture with a thin functional layer sandwiched between the fiber anode and outer cathode layer. Similar to planar OLEDs, OLED fibers show both high brightness and current efficiency due to efficient carrier injection and transformation from electrode to luminescent layer in the nanoscale thickness. Therefore, carrier-transporting layers and active metal electrodes with low work functions for matching energy levels are necessary for the OLED fibers, thus requiring complicated multilayered vacuum deposition processes with relatively low working stability. It remains challenging while critical to continuously produce OLED fibers for the next weaving process into textile displays.

Recently, PLED fibers have been developed because conjugated luminescent polymers display both high film-forming properties and flexibility [4]. Luminescent polymers can be loaded on fibers by solution processes like dip-coating, which is cost-effective and potential for continuous production. PLED fibers showed luminance over 10 000 cd/m^2^ under 10 V of bias and kept stable luminescence during manual weaving.

For LEC fibers, the emission layer can be either conjugated light-emitting polymers with polymer electrolyte and ionic salt [5] or ionic transition metal complexes [6]. It is sandwiched between two fiber electrodes. When an external bias is applied to the LEC fiber, the ion or electron migration in the emission layer leads to the formation of a p–i–n junction. The electrons and holes then move to the intermediate intrinsic junction and recombine to form excitons, resulting in light emission. Compared with OLED fibers, LEC fibers have lower requirements for energy-level matching of electrodes and smoothness of the substrate based on the mentioned robust and simple light-emitting mechanism. Therefore, a polymer LEC fiber can be continuously fabricated by sequentially dip-coating a luminescent layer on a conductive fiber electrode and wrapping a carbon nanotube sheet as transparent electrode outside [5]. The highest brightness reached is 950 cd/m^2^ with a current efficiency of 0.51 cd/A. Currently, the main efforts should be made to enhance the lifetime of the luminescent layer that is sensitive to water and oxygen.

For ACEL fiber, a light-emitting layer is made from luminescent powder dispersed in insulating polymer. By tuning the mechanical property of the insulating polymer, we may achieve a light-emitting layer with both high toughness and elasticity, which is more stable than OLED fibers. Although the brightness of the ACEL fiber is far lower than that of the existing OLED fiber (∼10 000 cd/m^2^), it can still exceed 200 cd/m^2^ at the electric field strength of 7.7 V/μm, which meets the requirement of indoor applications (100–300 cd/m^2^) [7]. Additionally, there is no need for charge transport at the electrode interface and only simple physical contact is required to achieve effective excitation. Therefore, a variety

of fiber-processing techniques, such as twisting, extrusion [7] and dip-coating [8], can be used to assemble ACEL fibers. It is exciting to see that these ACEL fibers have been already produced continuously to be compatible with currently standardized weaving processes of industrial textile machines, possibly paving the way for real applications of light-emitting fibers in the near future. More efforts are thus required to explore general and effective integration methods to construct textiles and further enhance both stability and safety of the resulting light-emitting textiles.

However, the light-emitting textiles woven from light-emitting fibers could only show very limited patterns. Although it is promising for many electronic products such as safety-alerting clothes for policemen and smart textiles for health monitoring, which just ask for a few patterns for displaying, they are not real displays as we expect. In our daily life, we use pixel displays for computers and cell phones, and they can produce continuous and dynamic information outputs. Based on the structure and property characteristics in nature, it is difficult to integrate such light-emitting fibers into pixel displays.

An interwoven strategy of luminescent fiber was designed to address this longstanding problem (Fig. 1c) [9]. The electroluminescent unit is constructed directly by the crossing of the luminescent warp and transparent conductive weft, which is similar to the arrangement of modern displays. Each electroluminescent unit thus can be individually controlled by the voltage difference between its warp and weft electrodes. The

transparent conductive wefts were spun from ionic liquid-polyurethane gel. The luminescent warps were silver-coated nylon yarns coated with a ZnS phosphor layer. A contact point creates a stable and uniform electric field between the warp and weft electrodes. Importantly, in mimicking the weaving strategy in the textile industry, it is easy to continuously produce such textile displays on a large scale. A 6-meter-long, 25-centimeter-wide textile display containing 5 × 10^5^ electroluminescent units had been achieved by this approach. Such textile displays are flexible and breathable, and they can withstand repeated machine-washing, making them suitable for practical applications such as navigation or healthcare.

For the above textile display, the luminescent intensities are typically low based on the use of ZnS phosphor. For instance, the brightness of outdoor display equipment usually ranges from 500 to 1000 cd/m^2^, which is difficult for the current luminescent ZnS materials, even driven by extremely high voltages. Therefore, it is natural to improve the brightness and color richness through the use of OLED technology [10,11]. Until now, the number of light-emitting points reported in the textile does not exceed 32 [11] because the OLED patterned on the fiber substrate requires high precisions of material depositions and weaving processes. Great efforts are still needed to prepare large-area textile displays with this strategy.

Besides the further improvement in the luminescent intensity, it is key to enhance the resolution of these textile displays. The particle sizes of ZnS luminescent powders are typically 20–50 μm. As a result, the available resolutions of tens of pixels per inch are much lower than expected for practical applications in most electronic products. A possible route that has been investigated is to construct microscopic light-emitting units at interwoven points [12]. In addition, it is recognized that full-color display is based on the combination of red, green and blue light-emitting units. However, there is a lack of efficient red color for luminescent ZnS materials.

To extend the applications of textile displays, it is further important to integrate them with the other functions of electronic textiles. The resulting textile display systems are demonstrated to be very useful for a spectrum of applications. On the one hand, it will promote the evolutionary development of existing information technology, lifting the restriction of wearables in small accessories such as smart watches, bands and glasses. On the other hand, textile displays are expected to create new industrial applications, such as precise diagnosis and self-monitoring in smart healthcare and intelligent home and office in the Internet of Things.

Although the revolutionary impacts of textile displays have been extensively investigated at both academy and industry, the optical performance of the interwoven textile display still has a long way to go before catching up with modern display technology. With the synthesis of new luminescent materials, design of novel weaving microstructures and discovery of promising integration methods, the resulting high-performance textile displays as the next-generation display technology may enter daily life and change the way we live, hopefully in the following 10–20 years.
